# Maillard Reaction Modification of Walnut Gluten Antioxidant Peptides: Process Optimization, Conformational Rearrangement, and Flavor Formation

**DOI:** 10.3390/foods15091520

**Published:** 2026-04-27

**Authors:** Yansong Gao, Zhiqiang Lu, Han Yang, Shanshan Liu, Lin Wang, Qiang Ma, Zhenchao La, MAMAN Baligen, Lingming Kong

**Affiliations:** 1 College of Food Science and Pharmacy, Xinjiang Agricultural University, Urumqi 830052, China; 19703538864@163.com (Y.G.);; 2 The Center for Disease Control and Prevention (Health Supervision Institute) of Midong District, Urumqi 831400, China; 3 Xinjiang Uyghur Autonomous Region Institute for Drug Control, Urumqi 830054, China

**Keywords:** walnut gluten peptides, Maillard reaction, process optimization, conformational change, flavor properties, antioxidant activity

## Abstract

To improve the flavor quality and antioxidant activity of walnut gluten peptides, gluten was extracted from defatted walnut meal by alkaline solubilization and acid precipitation, hydrolyzed with alkaline protease to prepare antioxidant peptides, and further modified by the Maillard reaction. The optimal sugar source was selected by single-factor experiments, and reaction conditions were optimized by response surface methodology. Peptide conformational changes were characterized by UV, fluorescence, DSC, FTIR, and SEM, while changes in amino acid composition, flavor properties, and antioxidant activity were systematically evaluated. Fructose was identified as the optimal sugar source. The optimal reaction conditions were a peptide-to-sugar ratio of 1:1.2, 78.5 °C, initial pH 7.6, and 2 h reaction time, under which the sensory score reached 8.5 and DPPH radical scavenging activity reached 66.92%. Maillard modification markedly altered peptide conformation, as shown by increased UV absorbance, decreased intrinsic fluorescence intensity with a red shift, an increase in denaturation temperature from 80 °C to 100 °C, reduced α-helix content, increased β-sheet content, and transformation of the microstructure from a loose porous morphology to dense block-like aggregates. Free amino acid content increased initially and then decreased, whereas total essential amino acids were largely retained, indicating that the overall nutritional composition was preserved. However, further evaluation of digestibility and bioavailability is required to confirm nutritional value. These findings provide a feasible strategy for improving the flavor and functional properties of walnut gluten peptides and support their high-value utilization.

## 1. Introduction

Walnut (*Juglans regia* L.) is an important woody oil crop and nut resource [1]. In industrial processing, large amounts of walnut kernels are used for oil production, generating substantial quantities of by-products, particularly defatted walnut meal [2]. Defatted walnut meal contains more than 40% protein, but its utilization remains limited because of bitterness and poor processing properties, including low solubility and weak functionality [3]. As a result, it is often used in low-value applications, leading to both resource waste and environmental burden. The development of defatted walnut meal into high-value protein ingredients or functional components is therefore of considerable interest for the walnut industry and for improving the nutritional value of walnut-derived products [4].

Alkaline extraction–isoelectric precipitation (AE–IP) is widely used for the isolation of plant proteins because of its simple procedure and low cost [5]. However, exposure to high pH and heat may induce protein unfolding, amino acid side-chain modifications, and aggregation, which can subsequently affect enzymatic hydrolysis efficiency and peptide functionality. In walnut protein systems, previous studies have shown that extraction and separation methods markedly influence protein composition, structure, and functional properties, highlighting the need to better control the relationship between processing conditions, structural changes, and functionality [6].

Enzymatic hydrolysis is an effective approach for producing small peptides with improved bioavailability and promising biological activities, and has therefore attracted increasing attention in functional food research [7]. Walnut-derived peptides are considered a potential source of natural antioxidants because of their essential amino acid content and antioxidant potential. Recent studies have described their preparation, bioactivity, and mechanisms of action, and some have further established bioactive peptide databases for walnut hydrolysates to identify key antioxidant peptides [8]. Despite this potential, the application of protein hydrolysates and peptide products is often limited by undesirable flavor. Enzymatic hydrolysis frequently generates hydrophobic peptides associated with bitterness, and bitterness intensity is closely related to hydrophobic amino acid exposure and molecular weight distribution [9].

The Maillard reaction (MR) is one of the most important non-enzymatic browning reactions in food systems. It involves a series of condensation, rearrangement, and polymerization reactions between the carbonyl groups of reducing sugars and the free amino groups of amino compounds [10]. Under controlled conditions, Maillard-type glycation has been widely reported to improve the processing properties of proteins and peptides, such as thermal stability and solubility, while also enhancing biological activities, including antioxidant capacity [11,12]. In addition, volatile compounds and intermediate- to late-stage reaction products generated during the Maillard reaction contribute substantially to aroma, taste, and color development. Reaction conditions, especially sugar type, sugar ratio, pH, time, and temperature, strongly affect reaction pathways, product profiles, structural remodeling, flavor generation, and antioxidant performance [13]. However, excessive Maillard reaction may also produce undesirable advanced glycation end products (AGEs), making precise control of reaction intensity essential. Previous studies using pea protein hydrolysates have shown that Maillard reaction intermediates can reduce bitterness and enhance umami and saltiness, demonstrating the potential of peptide–sugar reactions for flavor improvement in plant protein hydrolysates [14,15]. Nevertheless, for antioxidant peptides derived from walnut gluten, most studies have focused mainly on process optimization or activity evaluation. The mechanisms by which key parameters, particularly temperature, regulate reaction progression, structural remodeling, flavor development, and antioxidant enhancement remain insufficiently understood [16].

Compared to previous studies on plant protein hydrolysates, this work provides a novel, integrated perspective by: (1) systematically evaluating the effect of temperature on the progression of the Maillard reaction and its trade-off between flavor improvement and advanced glycation; (2) elucidating the relationship between peptide conformational rearrangement (from secondary structure to microstructure) and flavor/antioxidant enhancement; and (3) establishing a controllable modification strategy specifically for walnut gluten peptides.

Therefore, in this study, walnut gluten was isolated from defatted walnut meal and hydrolyzed to prepare antioxidant peptides. After screening the sugar source, a process optimization model for Maillard modification was established, with particular emphasis on the effect of temperature on reaction progression. Structural evolution and aggregation behavior were further characterized by UV spectroscopy, intrinsic fluorescence spectroscopy, FTIR, DSC, and SEM. Changes in free and hydrolyzed amino acid composition, flavor properties, and antioxidant activity were also systematically evaluated. This study aimed to establish a controllable Maillard modification strategy for walnut gluten peptides and to clarify the relationship among structural changes, flavor improvement, and functional enhancement, thereby providing theoretical support and technical guidance for the high-value utilization of walnut processing by-products.

## 2. Materials and Methods

### 2.1. Materials and Reagents

Dehulled defatted walnut meal with a protein content of 58.3% was purchased from Yecheng Shenlan Technology Co., Ltd. (Yecheng County, Xinjiang, China) and used as the raw material. Alkaline protease (Catalog No.: S10154, Activity: 200 U/mg) was obtained from Shanghai Yuanye Biotechnology Co., Ltd. (Shanghai, China). Glucose (AR, ≥99.5%), fructose (AR, ≥99.0%), xylose (AR, ≥98.5%), arabinose (AR, ≥99.0%), sodium bicarbonate, citric acid, disodium hydrogen phosphate, potassium dihydrogen phosphate, sodium hydroxide, hydrochloric acid, absolute ethanol, and sulfosalicylic acid were of analytical grade and purchased from Sinopharm Chemical Reagent Co., Ltd. (Shanghai, China). DPPH (2,2-diphenyl-1-picrylhydrazyl, purity ≥ 97%) was purchased from Sigma-Aldrich (St. Louis, MO, USA). Amino acid standards (purity ≥ 98%) were purchased from Sigma-Aldrich (St. Louis, MO, USA). High-purity nitrogen (purity ≥ 99.999%) was supplied by Beijing Helipu Beifen Gas Industry Co., Ltd. (Beijing, China). Deionized water was prepared in the laboratory. All other chemicals and solvents used in this study were of analytical grade with purity ≥ 98.0% unless otherwise specified.

### 2.2. Instruments and Equipment

A UV 1200 UV visible spectrophotometer was obtained from Shanghai Meipuda Instrument Co., Ltd. (Shanghai, China). An F 7000 fluorescence spectrophotometer was purchased from Hitachi (Tokyo, Japan). A DSC200F3 differential scanning calorimeter was purchased from Netzsch (Selb, Germany). A Nicolet iS50 Fourier transform infrared spectrometer was obtained from Thermo Fisher Scientific (Waltham, MA, USA). An SU8010 scanning electron microscope and an L 8900 automatic amino acid analyzer were purchased from Hitachi (Tokyo, Japan). A FlavourSpec gas chromatography ion mobility spectrometer was obtained from G.A.S. (Dortmund, Germany). A 7890B 5977A gas chromatography mass spectrometry system was purchased from Agilent Technologies (Santa Clara, CA, USA). An Easy nLC 1200 coupled with a Q Exactive HF liquid chromatography tandem mass spectrometry system was obtained from Thermo Fisher Scientific (Waltham, MA, USA). A CTFD 10 12 18 U/UPlus vacuum freeze dryer was purchased from Qingdao Yonghe Innovation Electronic Technology Co., Ltd. (Qingdao, Shandong, China). The following instruments were used in this study. A PHS-3C pH meter (Shanghai LeiCi Instruments Co., Ltd., Shanghai, China), an LXJ-IIB centrifuge (Shaanxi Huanyu Instrument Equipment Co., Ltd., Xi’an, China), and a KD-810C automated microplate reader (Beijing Purkinje General Instrument Co., Ltd., Beijing, China) were obtained from domestic suppliers. An HH S constant temperature water bath (Shanghai Jinghong Experimental Equipment Co., Ltd., Shanghai, China), a TDL-5A centrifuge (Shanghai Anting Scientific Instrument Factory, Shanghai, China), and an FA1204B electronic analytical balance (Shanghai Liangping Instrument Co., Ltd., Shanghai, China) were also used. A CM-5 colorimeter (Konica Minolta, Tokyo, Japan) was employed for color measurements. All other common laboratory consumables were of analytical grade.

### 2.3. Preparation of Walnut Gluten Peptide

Defatted walnut meal was mixed with deionized water (1:40, *w*/*v*), adjusted to pH 12, and stirred at 55 °C for 90 min. After centrifugation (5000× *g*, 20 min), the supernatant was adjusted to pH 4.5, stirred for 1 h, and centrifuged again (5000× *g*, 20 min). The precipitate was freeze-dried to obtain crude walnut protein.

The crude protein was fractionated using the Osborne method [17]. Deionized water, NaCl solution, ethanol solution, and NaOH solution were sequentially used to extract albumin, globulin, prolamin, and gluten, respectively. The gluten fraction was collected and freeze-dried.

Walnut gluten was then hydrolyzed with alkaline protease (enzyme dosage 5000 U/g, pH 8.0, 55 °C, 4 h) according to Liu et al. [18]. The enzyme was inactivated (boiling water bath, 10 min), and the hydrolysate was centrifuged (4500× *g*, 20 min). The supernatant was filtered and subjected to ultrafiltration using a 3 kDa membrane. The permeate was freeze-dried to obtain walnut gluten peptides (WGPs).

### 2.4. Preparation of Maillard Modified Peptides

Following Jin et al. [19], WGP and reducing sugar were dissolved in phosphate buffer (peptide concentration 50 g/L) at the desired ratio. The pH was adjusted, and the solution was incubated in the dark at a set temperature. The reaction was stopped by cooling in an ice bath. The mixture was dialyzed (1000 Da cut-off) for 24 h to remove unreacted sugars, then freeze-dried to obtain the fructose-modified peptide (FMP).

### 2.5. Response Surface Optimization

Preliminary single-factor experiments were first conducted to determine the optimal sugar source and appropriate parameter ranges for response surface methodology (RSM). Under fixed conditions (peptide:sugar ratio 1:1, pH 7.0, 70 °C, 2 h), the effects of sugar type (glucose, fructose, xylose, arabinose) were tested. Subsequently, the effects of temperature (60–90 °C), initial pH (6.0–9.0), and peptide:sugar ratio (1:0.5–1:2) were evaluated. Based on these results, a three-factor, three-level Box–Behnken design (BBD) was used to optimize temperature (A), initial pH (B), and peptide:sugar ratio (C) with sensory score (Y_1_) and DPPH scavenging activity (Y_2_) as responses (Table 1).

### 2.6. Color Measurement

The method of Lan et al. [20] was used with slight modifications. A 2 mL sample was transferred into a Petri dish with a diameter of 3 cm. Distilled water was used as the reference, and the color parameters ΔL*, Δa*, and Δb* were measured using a colorimeter, representing lightness, red–green value, and yellow–blue value, respectively. The total color difference ΔE was calculated as follows:

ΔE = (ΔL^2^ + Δa^2^ + Δb*^2^)^1/2^

All measurements were performed in triplicate, and the mean value was reported.

### 2.7. Determination of Maillard Reaction Intermediate

The method of Li et al. [21] was used with slight modifications. One milliliter of sample supernatant was diluted tenfold with distilled water, and the absorbance at 294 nm was measured using a microplate reader. The absorbance value at 294 nm was used to indicate the relative content of Maillard reaction intermediates. All measurements were performed in triplicate, and the mean value was reported.

### 2.8. Browning Index

The method of Wang et al. [22] was used with slight modifications. One milliliter of sample supernatant was diluted tenfold with distilled water, and the absorbance at 420 nm was measured using a microplate reader. The browning index was calculated using the following equation:

Browning index = (A_420_ of sample minus A_420_ of blank) × 10

All measurements were performed in triplicate, and the mean value was reported.

### 2.9. Sensory Evaluation

Sensory evaluation was performed with reference to ISO 6658:2017 [23], ISO 8586:2023 [24], ISO 8589:2007 [25], ISO 13299:2016 [26], and ISO 4121:2003 [27], with slight modifications according to the sample characteristics. The sensory panel consisted of 10 screened and trained assessors. Training included recognition of standard flavor compounds, with 2 methyl 3 furanthiol used as the reference compound for meaty aroma and 2,5 dimethylpyrazine used as the reference compound for roasted aroma.

The evaluation was conducted in individual sensory booths that met the requirements of ISO 8589 [25]. A 5 mL aliquot of sample supernatant was placed in a transparent glass cup. Each assessor sequentially evaluated aroma and taste and recorded the intensity using a quantitative scoring scale (Table 2). Between samples, assessors rinsed their mouths with purified water for 30 s to minimize carryover effects. Results were expressed as the mean score of the 10 assessors. The final sensory score was calculated by assigning equal weights to aroma and taste, with each accounting for 50%. The samples were presented to the panelists in a randomized and blind fashion. Each sample was evaluated in triplicate. Panel performance was monitored for consistency.

### 2.10. DPPH Radical Scavenging Activity

The method of Benjakul et al. [28] was used with slight modifications. A DPPH solution at a concentration of 2 mmol/L was prepared in absolute ethanol and mixed thoroughly with the modified peptide solution at a volume ratio of 1:1. This concentration has been widely used for evaluating DPPH scavenging of Maillard reaction products to ensure sufficient radical excess for comparison among samples. After incubation in the dark at room temperature for 30 min, the absorbance was measured at 517 nm. Absolute ethanol was used as the reference. For the blank control, deionized water was used instead of the modified peptide solution, and the absorbance was measured under the same conditions. The DPPH radical scavenging activity was calculated according to the following equation:

DPPH free radical scavenging rate = [A_0_ − (A_1_ − A_2_)]/A_0_ × 100%

where A_1_ is the absorbance of the mixture of modified peptide solution and DPPH ethanol solution, A_2_ is the absorbance of the mixture of modified peptide solution and absolute ethanol, and A_0_ is the absorbance of the mixture of absolute ethanol and DPPH ethanol solution.

### 2.11. UV Absorption Spectroscopy

The method of Ajandouz et al. [29] was used with slight modifications. A peptide solution with a concentration of 0.1 mg/mL was prepared. Phosphate buffer at 0.01 mol/L and pH 7.0 was used as the blank control. The absorption spectrum was recorded using a UV 1200 UV visible spectrophotometer over the wavelength range from 200 to 400 nm with an interval of 1 nm.

### 2.12. Intrinsic Fluorescence Spectroscopy

The method of Morales et al. [30] was used with slight modifications. The peptide solution concentration was adjusted to 0.1 mg/mL. The excitation wavelength was set at 370 nm, and both the excitation slit and emission slit were set at 5 nm. The fluorescence emission spectrum was scanned from 390 to 500 nm using an F 7000 fluorescence spectrophotometer, and the fluorescence intensity was recorded.

### 2.13. Differential Scanning Calorimetry Analysis

The method of Liu et al. [31] was used with slight modifications. A 5 mg lyophilized sample was accurately weighed and placed in an aluminum crucible. An empty crucible was used as the reference. Under a nitrogen atmosphere with a flow rate of 50 mL/min, the sample was heated from 25 °C to 175 °C at a rate of 10 °C/min, and the heat flow versus temperature curve was recorded.

### 2.14. Fourier Transform Infrared Spectroscopy

The method of Carbonaro et al. [32] was used with slight modifications. The lyophilized sample was mixed thoroughly with KBr at a mass ratio of 1:100 and ground evenly. After tableting, the sample was scanned using a Nicolet iS50 Fourier transform infrared spectrometer over the range from 4000 to 400 cm^−1^, with a resolution of 4 cm^−1^ and 32 scans.

### 2.15. Scanning Electron Microscopy

The method of Kutzli et al. [33] was used with slight modifications. A small amount of lyophilized peptide sample was evenly spread onto conductive adhesive tape and sputter coated with gold for 30 s at 15 mA. The sample was then observed using an SU8010 scanning electron microscope at an accelerating voltage of 5 kV and a working distance of 9.8 to 9.9 mm, with a magnification of 500 times. The microstructure and aggregation state were recorded.

### 2.16. Free Amino Acid Composition Analysis

The method of Ming et al. [18] was used with slight modifications. A 20 mg sample was accurately weighed, and 10 mL of 5% sulfosalicylic acid solution was added. The mixture was vortexed for 30 min, allowed to stand overnight at 4 °C, and then centrifuged at 10,000× *g* for 20 min. The supernatant was collected and filtered through a 0.22 μm membrane, followed by analysis using an L 8900 automatic amino acid analyzer. The contents of 21 free amino acids were determined, and the total free amino acid content was calculated.

### 2.17. GC IMS Analysis

The method of Yi et al. [34] was used with slight modifications. One milliliter of sample was transferred into a 20 mL headspace vial and incubated at 60 °C for 15 min, followed by automatic injection of 500 μL. Volatile compounds were analyzed using a FlavourSpec GC IMS system equipped with an FS SE 54 CB 1 capillary column. Nitrogen was used as both the carrier gas and drift gas. The carrier gas flow rate was programmed from 0 to 100 mL/min, and the drift gas flow rate was set at 150 mL/min. The drift tube temperature was 45 °C, and the tube length was 98 mm. Characteristic flavor fingerprints were identified using GC IMS Library 2.0, and differences among samples were analyzed using the Reporter plug in.

### 2.18. GC MS Analysis

The method of Zeng et al. [35] was used with slight modifications. A 5 mL sample was transferred into a 15 mL headspace vial, and 2 μL of 1,2 dichlorobenzene was added as the internal standard. A 50/30 μm DVB/CAR/PDMS solid phase microextraction fiber was used for headspace extraction in a 55 °C water bath for 30 min, followed by thermal desorption at the GC injection port at 250 °C for 3 min.

Volatile compounds were analyzed using an Agilent 7890B 5977A gas chromatography mass spectrometry system equipped with a DB WAX capillary column. Helium was used as the carrier gas at a flow rate of 1.8 mL/min, and splitless injection was applied. The oven temperature program was as follows: the initial temperature was held at 40 °C for 3 min, increased to 80 °C at 5 °C/min, increased to 160 °C at 10 °C/min and held for 0.5 min, increased to 175 °C at 2 °C/min, and finally increased to 230 °C at 10 °C/min and held for 7 min. Electron impact ionization was performed at 70 eV. The ion source temperature was 250 °C, the interface temperature was 250 °C, and the scan range was *m*/*z* 35 to 450. Qualitative identification was carried out by calculating the retention indices of C6 to C26 normal alkanes and comparing them with the NIST 20 database.

### 2.19. Data Analysis

All experiments were performed in triplicate, and the results are expressed as mean ± standard deviation. Analysis of variance was performed using SPSS 22.0, and differences were considered significant at *p* < 0.05. Response surface design and data analysis were carried out using Design Expert 8.0.6. Figures were generated using Origin 2024.

## 3. Results

### 3.1. Effects of Different Monosaccharides on Sensory Properties, Antioxidant Activity, and Reaction Progression of Maillard Modified Walnut Gluten Peptides

**Figure 1:**
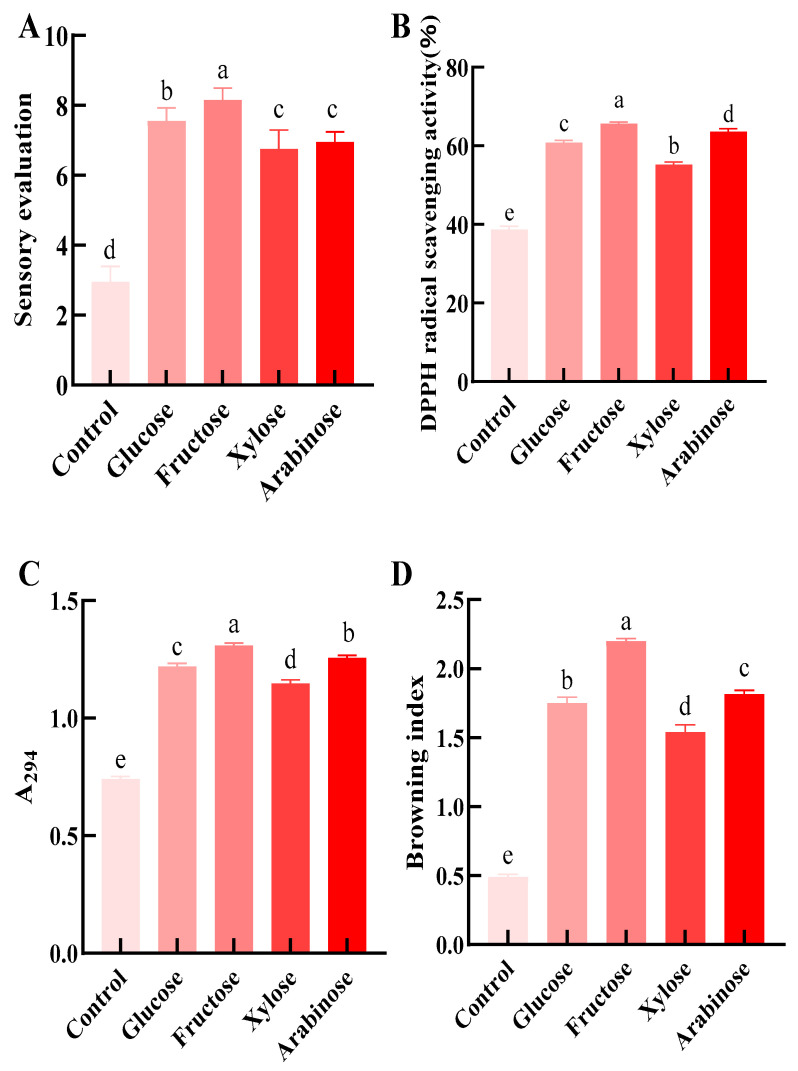
Effects of different monosaccharides on the sensory, antioxidant and reaction process parameters of Maillard-modified walnut gluten peptides. (**A**) Sensory scores; (**B**) DPPH radical scavenging activity; (**C**) A_294_ value; (**D**) Browning index. Data are presented as mean ± SD (*n* = 3). Different lowercase letters (a, b, c, d, e) indicate significant differences among groups (*p* < 0.05).

At a peptide to sugar ratio of 1:1, initial pH of 7.0, 70 °C, and 2 h, glucose, fructose, xylose, and arabinose were compared for Maillard modification of walnut gluten peptides [14,36]. As shown in Figure 1A,B, all sugar systems significantly increased sensory score and DPPH radical scavenging activity compared with the control group (*p* < 0.05). Fructose showed the best overall performance, with a sensory score of 8.1 and a DPPH radical scavenging activity of 66%. Glucose ranked second, with values of 7.5 and 60%, respectively. Xylose showed relatively high antioxidant activity at 63%, but its sensory score was only 6.7. Arabinose showed the weakest improvement, with a sensory score of 6.9 and a DPPH radical scavenging activity of 55%.

As shown in Figure 1C,D, A294 and the browning index were significantly higher in all monosaccharide systems than in the control group (*p* < 0.05), and both were highest in the fructose group. A294 reflects the accumulation of intermediate Maillard products, whereas browning index indicates the formation of late-stage brown pigments [37]. The higher values observed in the fructose group suggest a more active reaction process.

Overall, fructose produced the best results in reaction progression, sensory quality, and antioxidant activity. This may be related to its higher initial glycation reactivity in some food systems [38,39]. Therefore, fructose was selected as the optimal sugar source for subsequent response surface optimization and mechanistic analysis.

### 3.2. Effects of Temperature, pH, and Peptide to Sugar Ratio on Sensory Score, DPPH Radical Scavenging Activity, A294 Absorbance, and Browning Index of Fructose-Modified Peptides

**Figure 2:**
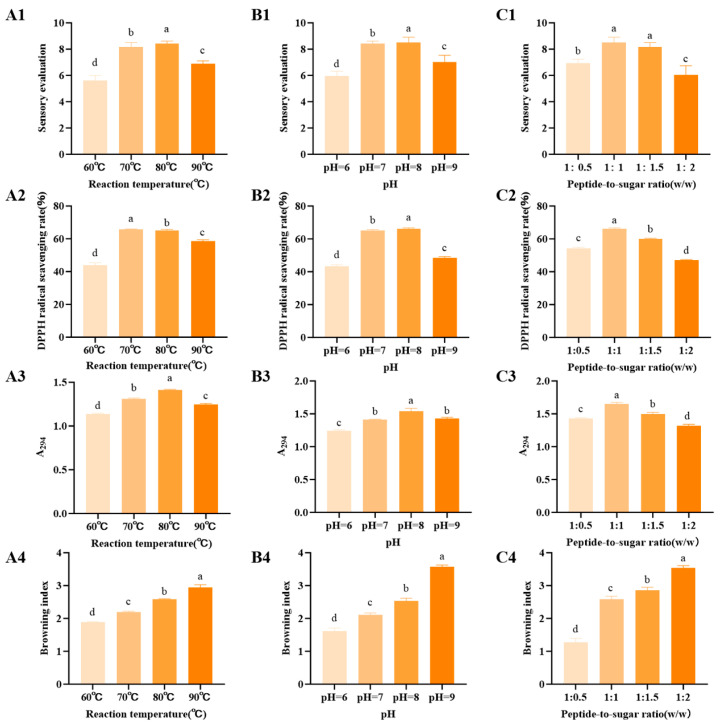
Effect of reaction conditions on the sensory scores, DPPH radical scavenging activity, A_294_ and browning index of Maillard reaction products from walnut gluten peptides. (**A1**–**A4**) Effect of reaction temperature on sensory score, DPPH radical scavenging activity, A_294_ and browning index. (**B1**–**B4**) Effect of pH on sensory score, DPPH radical scavenging activity, A_294_ and browning index. (**C1**–**C4**) Effect of peptide-to-sugar ratio on sensory score, DPPH radical scavenging activity, A_294_ and browning index. Data are presented as mean ± SD (*n* = 3). Different lowercase letters (a, b, c, d) indicate significant differences among groups (*p* < 0.05).

Temperature is a key factor affecting the rate and product composition of the Maillard reaction. Increasing temperature accelerates carbonyl amino condensation, rearrangement, cleavage, and polymerization, thereby altering the accumulation of reaction intermediates and browning products and affecting aroma and antioxidant properties [40]. As shown in Figure 2(A1,A2), when temperature increased from 60 °C to 80 °C, both sensory score and DPPH radical scavenging activity of fructose-modified peptides increased significantly (*p* < 0.05), from 5.6 and 43% to 8.4 and 65%, respectively. However, when temperature further increased to 90 °C, these values decreased to 6.9 and 58%, respectively. This suggests that moderately high temperature is more favorable for improving both flavor quality and antioxidant activity. Shakoor et al. [10] reported that Maillard reaction intermediates and some late-stage products can enhance antioxidant activity through hydrogen donation, electron donation, and metal chelation, while also contributing to flavor formation. However, an excessively high temperature may promote advanced polymerization, generate burnt flavor, and consume or transform some intermediates, thereby reducing sensory quality and antioxidant performance.

As shown in Figure 2(A3,A4), A294 increased significantly from 60 °C to 80 °C (*p* < 0.05) and reached a maximum value of 1.41 at 80 °C, then decreased to 1.24 at 90 °C. In contrast, browning index continued to increase with temperature and reached its highest value of 3.0 in the 90 °C group (*p* < 0.05). Tu et al. [41] reported that A294 reflects intermediates formed during the intermediate stage of the Maillard reaction, whereas absorbance at 420 nm reflects the accumulation of late-stage browning products. Therefore, the decline in A294 and continued increase in browning index at 90 °C indicate that high temperature promoted the conversion of intermediates into late-stage browning products.

pH regulates the Maillard reaction by affecting amino group protonation and the formation pathway of key intermediates. Neutral to mildly alkaline conditions can accelerate the reaction, whereas excessively alkaline conditions may lead to flavor deterioration and excessive browning [42]. As shown in Figure 2(B1,B2), when pH increased from 6 to 8, sensory score and DPPH radical scavenging activity increased significantly (*p* < 0.05). When pH further increased to 9, these values decreased to 7.0 and 48%, respectively. Previous studies have shown that pH exerts both promoting and adverse effects on flavor development. Neutral to mildly alkaline conditions are generally favorable for flavor formation, whereas excessively high pH may accelerate side reactions and the accumulation of late-stage products, thereby generating off flavor and reducing sensory quality. As shown in Figure 2(B3,B4), A294 increased significantly from pH 6 to pH 8 and reached 1.54 (*p* < 0.05), then decreased to 1.43 at pH 9. In contrast, browning index increased significantly with pH and reached its highest value of 3.4 at pH 9 (*p* < 0.05). These results indicate that increasing pH promoted the reaction from the intermediate stage to the late stage, and that pH 9 favored the accumulation of late-stage browning products.

The reactant ratio determines the effective collision probability between carbonyl groups and amino groups in the system, thereby influencing glycation degree, volatile flavor formation, and browning polymerization behavior [20]. As shown in Figure 2(C1,C2), when the peptide to sugar ratio increased from 1:0.5 to 1:1, sensory score and DPPH radical scavenging activity increased significantly (*p* < 0.05). The highest values were obtained at 1:1, reaching 8.5 and 65%, respectively. When the ratio further increased to 1:1.5, sensory score remained relatively high at 8.1, whereas DPPH radical scavenging activity decreased to 60%. At 1:2, both values decreased markedly to 6.0 and 47%, respectively. These results indicate that a moderate peptide to sugar ratio is more favorable for balancing flavor quality and antioxidant activity, whereas excess sugar may drive the reaction toward excessive browning or polymerization, thereby increasing the risk of burnt flavor and reducing product quality [10]. As shown in Figure 2(C3,C4), A294 increased significantly from 1:0.5 to 1:1 (*p* < 0.05) and reached 1.65 at 1:1, then decreased to 1.49 and 1.32 at 1:1.5 and 1:2, respectively. In contrast, browning index increased overall with increasing sugar proportion and reached its highest value of 3.6 in the 1:2 group (*p* < 0.05). Tu et al. [41] reported that A294 and browning index reflect products formed at different reaction stages. An appropriate sugar level favored the accumulation of intermediate products, whereas excessive sugar promoted the accumulation of late-stage browning products and the consumption of intermediates.

Overall, 70 to 80 °C, pH 7 to 8, and a peptide to sugar ratio of 1:1 were more favorable for maintaining high sensory score, strong DPPH radical scavenging activity, high A294, and moderate browning. In contrast, excessive temperature, alkalinity, or sugar level promoted excessive browning and reduced product quality. Therefore, optimization of the Maillard reaction should aim to balance sufficient formation of intermediate products with moderate accumulation of late-stage browning products.

### 3.3. Results of Response Surface Experiments and Verification of the Optimal Conditions for Temperature, Initial pH, and Peptide to Sugar Ratio

The Maillard reaction conditions were optimized using response surface methodology. As shown in Table 3, A Box–Behnken design with three factors and three levels was applied, resulting in 17 experimental runs. Reaction temperature (A), initial pH (B), and peptide to sugar ratio (C) were selected as the independent variables, and sensory score (Y_1_) and DPPH radical scavenging activity (Y_2_) were used as the response values. Quadratic regression equations were established for both responses.

Analysis of variance showed that both models were highly significant. The F values of the sensory score model and the DPPH radical scavenging activity model were 30.34 and 240.65, respectively (*p* < 0.0001). The lack of fit was not significant, with *p* values of 0.0737 and 0.0632, indicating good agreement between the regression models and the experimental data. As shown in Table 4, The R^2^ and adjusted R^2^ values were 0.9750 and 0.9429 for sensory score, and 0.9968 and 0.9926 for DPPH radical scavenging activity, respectively, showing that the models explained more than 97% of the variation in the responses. The coefficients of variation were 2.39% and 1.01%, and the signal to noise ratios were 16.91 and 48.01, respectively, further confirming the high precision and reliability of the models.

**Figure 3:**
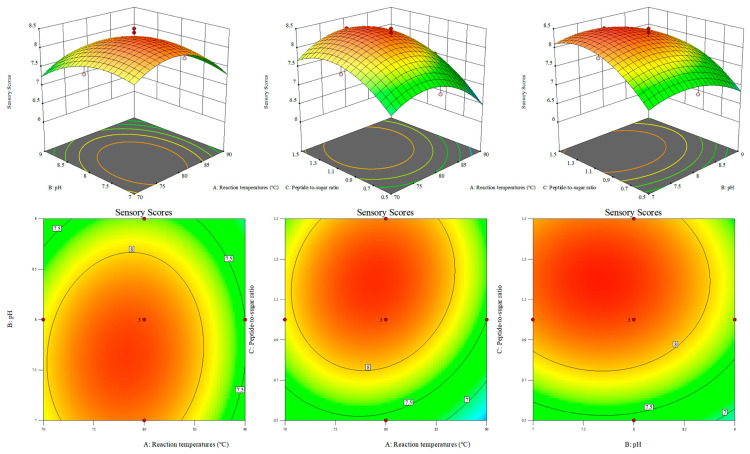
Response surface plots and contour plots of sensory scores for the interactions between experimental factors.

**Figure 4:**
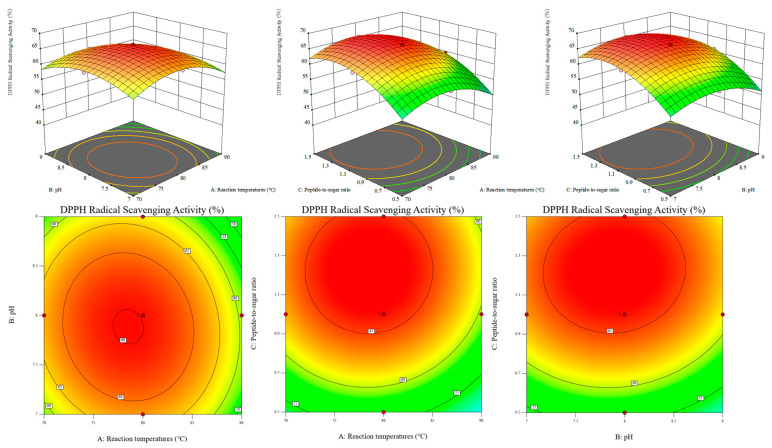
Response surface and contour plots of DPPH radical scavenging efficiency as a function of the interaction between experimental factors.

As shown in Figure 3 and Figure 4, Response surface analysis showed that all three factors had highly significant effects on sensory score and DPPH radical scavenging activity (*p* < 0.01). For sensory score, the order of influence was peptide to sugar ratio, initial pH, and reaction temperature. For DPPH radical scavenging activity, the order was peptide to sugar ratio, reaction temperature, and initial pH. Numerical optimization using Design Expert gave the optimal conditions as a reaction temperature of 78.5 °C, an initial pH of 7.6, and a peptide to sugar ratio of 1:1.2, with predicted values of 8.434 for sensory score and 66.815% for DPPH radical scavenging activity.

The reliability of the model was verified by three parallel experiments. The average sensory score and DPPH radical scavenging activity of the modified peptide were 8.5 and 66.92%, respectively, with relative errors of 0.78% and 0.16%, indicating good predictive accuracy. As shown in Table 5, under the optimal conditions, the fructose-modified walnut gluten peptide, abbreviated as FMP, showed an A294 value of 1.22 ± 0.01 and a browning index of 0.21 ± 0.01, which were 58.4% and 320% higher than those of the unmodified walnut gluten peptide, abbreviated as WGP. The total color difference ΔE* of FMP was 15.07 ± 0.06, markedly higher than the 1.59 ± 0.11 of WGP. These results indicate that both intermediate and late-stage Maillard reaction products were effectively generated under the optimized conditions, and that the reaction proceeded sufficiently without obvious over-browning, consistent with the response surface optimization results.

### 3.4. Effects of the Maillard Reaction on the UV Spectra of Walnut Gluten Peptides

**Figure 5:**
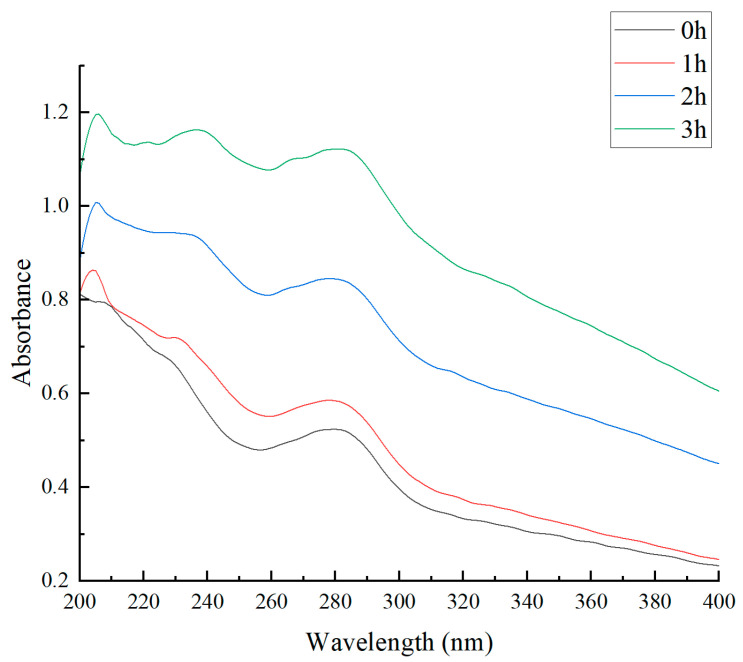
UV absorption spectra of Maillard-modified walnut gluten peptides at different reaction times.

UV absorption spectroscopy is an effective tool for monitoring protein structural changes and product formation during the Maillard reaction [43]. As shown in Figure 5, with increasing reaction time, the absorbance of Maillard-modified walnut gluten peptides showed an overall increase throughout the 200 to 400 nm range, reflecting progressive structural changes in the peptides and the gradual formation of characteristic Maillard reaction products.

In the 200 to 240 nm region, absorbance increased markedly. At 200 nm, the absorbance rose from 0.80 to 1.15, an increase of 43.75%. Absorption in this region is mainly attributed to peptide bond transitions and aromatic amino acid chromophores. The continuous increase suggests that the Maillard reaction might have promoted partial unfolding of the peptide structure, potentially leading to greater exposure of buried aromatic residues to the aqueous environment. In addition, early glycation derivatives and small molecular aldehydes formed during the reaction may also contribute to absorption in this region.

In the 220 to 280 nm region, absorbance also increased gradually with reaction time. For example, the absorbance at 260 nm increased from 0.50 to 1.08, more than doubling. This region corresponds to the characteristic absorption of proteins and Maillard reaction intermediates. Absorption near 260 nm is often associated with conjugated carbonyl compounds, whereas 280 nm is the characteristic absorption region of tyrosine and tryptophan. After 3 h of reaction, the absorbance at 280 nm reached 1.05, about 102% higher than that at 0 h, indicating that the microenvironment of peptide chromophores changed during the reaction and that reaction products containing aromatic or conjugated structures gradually accumulated.

In the 300 to 400 nm region, absorbance likewise showed a clear upward trend. At 340 nm, it increased from 0.35 to 0.82. Late-stage Maillard reaction products usually possess longer conjugated systems and more complex polymeric structures, which can extend absorption toward the visible region. Wang et al. [22] reported that melanoidin-like substances still show absorption at 420 nm and largely determine the brown appearance of the system. Therefore, the increase at 340 nm may reflect the gradual formation of late-stage products with extended conjugated structures. If combined with 420 nm absorbance or browning index data, this change would provide stronger evidence for linking the increase at 340 nm with the degree of final browning polymerization.

### 3.5. Effects of the Maillard Reaction on the Intrinsic Fluorescence Spectra of Walnut Gluten Peptides

**Figure 6:**
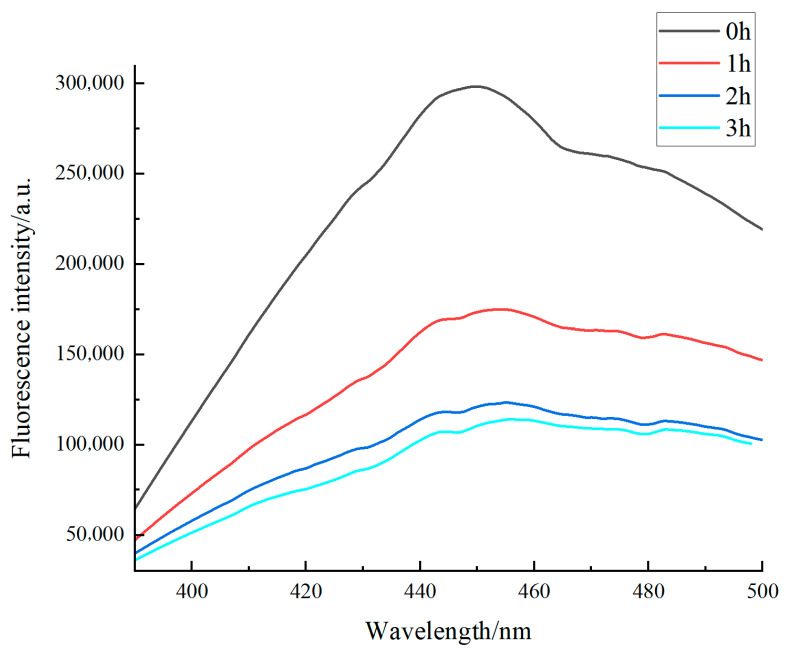
Fluorescence spectra of Maillard-modified walnut gluten peptides at different reaction times.

Intrinsic fluorescence spectroscopy is an effective method for monitoring changes in the tryptophan microenvironment during the Maillard reaction [44]. As shown in Figure 6, the unreacted sample showed the highest fluorescence intensity, with a maximum emission peak at 450 nm of about 300,000 a.u. As reaction time increased, fluorescence intensity decreased progressively. After 1 h, the intensity decreased to about 180,000 a.u., and the maximum emission wavelength shifted from 450 to 455 nm. After 2 h, the intensity further decreased to about 120,000 a.u., with the maximum emission wavelength shifting to 460 nm. After 3 h, the fluorescence intensity remained at about 100,000 a.u., and the maximum emission wavelength stayed at 460 nm.

The high fluorescence intensity of the unreacted sample is consistent with tryptophan residues being relatively exposed to a hydrophilic microenvironment. After 1 h, the marked decrease in fluorescence intensity and the 5 nm red shift suggested that glycation had begun and that the microenvironment of some tryptophan residues had changed. After 2 h, the further decrease in fluorescence intensity and continued red shift indicated stronger peptide sugar interactions and progressive conformational rearrangement, with tryptophan residues likely located in a more hydrophobic microenvironment, leading to enhanced fluorescence quenching.

After 3 h, only slight changes in fluorescence intensity were observed, and the maximum emission wavelength remained unchanged, indicating that the reaction gradually approached equilibrium and that the microenvironment of fluorescent groups became relatively stable. In addition, late-stage Maillard reaction products, such as melanoidin-like substances, may further weaken the fluorescence signal through the inner filter effect. Overall, the fluorescence results confirm the progressive development of the Maillard reaction and indicate a shift in peptide conformation and tryptophan microenvironment from relatively hydrophilic to more hydrophobic conditions.

### 3.6. Effect of the Maillard Reaction on the Thermal Stability of Walnut Gluten Peptides

**Figure 7:**
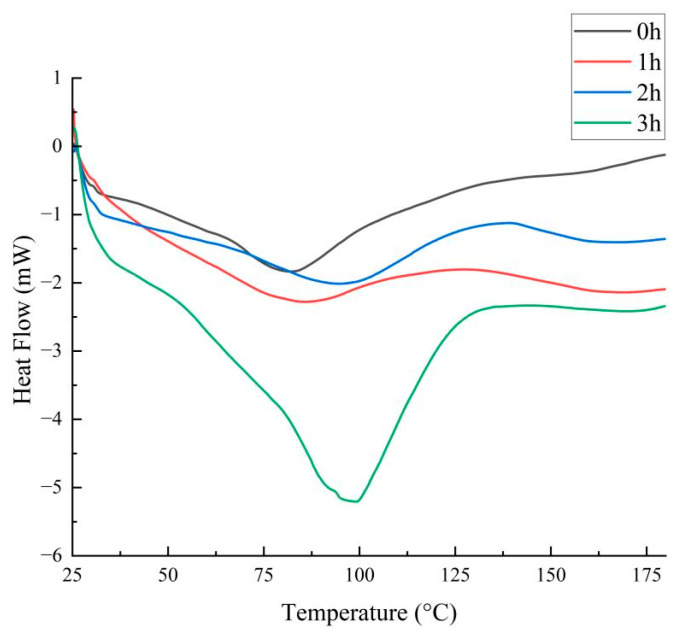
Differential scanning calorimetry of Maillard-modified walnut gluten peptides at different reaction times.

Differential scanning calorimetry was used to evaluate the effect of the Maillard reaction on the thermal stability of walnut gluten peptides [45]. As shown in Figure 7, the unmodified sample exhibited a broad and weak endothermic peak at about 80 °C, with a heat flow of about negative 1.7 mW, indicating a relatively loose peptide conformation and low thermal stability. After 1 h of reaction, the endothermic peak shifted to about 85 °C and the heat flow increased to about negative 2.2 mW, suggesting that early glycation began to stabilize peptide conformation and improve resistance to thermal denaturation.

After 2 h, the endothermic peak further shifted to 92 °C, with a heat flow of about negative 2.0 mW, and the peak became broader. The increase in transition temperature indicates further formation of peptide sugar crosslinking structures, which improved overall thermal stability, whereas peak broadening suggests some structural heterogeneity at this stage. After 3 h, the most pronounced change was observed, with a strong endothermic peak appearing at 100 °C and the heat flow increasing markedly to about negative 5.5 mW. This result indicates that the Maillard reaction had progressed substantially and formed a denser and more stable crosslinked network.

Overall, the increase in transition temperature and heat flow confirms that Maillard modification markedly improved the thermal stability of walnut gluten peptides.

### 3.7. Effects of the Maillard Reaction on the Secondary Structure of Walnut Gluten Peptides

Protein secondary structure is the basis of higher order conformation and functional properties, mainly including α helix, β sheet, β turn, and random coil. Fourier transform infrared spectroscopy is an effective method for analyzing these structures, and the amide I band at 1700 to 1600 cm^−1^ is particularly important [46]. This band mainly arises from C=O stretching vibration in the peptide backbone, and its frequency is strongly affected by the surrounding hydrogen bonding environment [47]. Therefore, changes in peak shape and position in the amide I region can reflect alterations in protein secondary structure.

**Figure 8:**
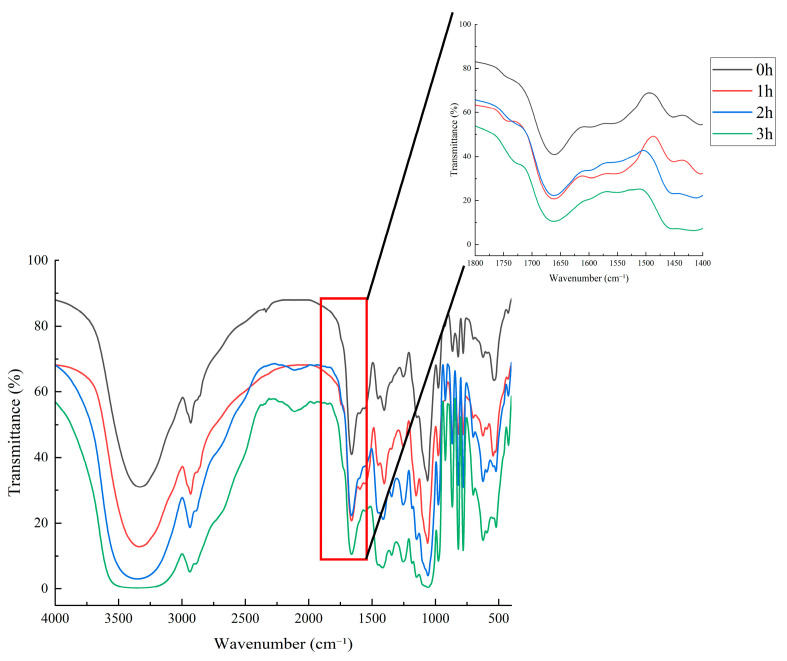
Fourier transform infrared spectra of Maillard-modified walnut gluten peptides at different reaction times.

Figure 8 shows the effects of different Maillard reaction times, 0, 1, 2, and 3 h, on the infrared spectra of walnut gluten peptides. In the amide I region, the main absorption peak near 1650 cm^−1^ gradually broadened as reaction time increased from 0 to 3 h, with a slight shift toward lower wavenumbers. At the same time, peak asymmetry increased and the low wavenumber side became broader. Compared with the other samples, the unreacted sample at 0 h showed the sharpest and most symmetrical amide I band. With increasing reaction time, the progressive broadening and red shift of the band indicate that the chemical microenvironment of C=O groups became more diverse and heterogeneous. These changes suggest that the hydrogen bond network maintaining the original ordered structure was weakened or rearranged, leading to reduced conformational rigidity and increased structural disorder.

As shown in Table 6, the secondary structure composition of walnut gluten peptides also changed during the Maillard reaction. In the unmodified sample at 0 h, β turn was the dominant structure at 43.55%, followed by α helix at 21.77% and β sheet at 20.08%, whereas random coil showed the lowest content at 14.60%. After 1 h, α helix and β sheet remained nearly unchanged, while β turn decreased slightly to 42.62% and random coil increased to 15.61%. After 2 and 3 h, α helix decreased to 20.11% and 20.03%, respectively, whereas β sheet increased to 21.92% and 22.57%. β turn decreased to 42.12% at 3 h, and random coil changed slightly to 15.29%.

Overall, with increasing Maillard reaction time, α helix content decreased slightly, β sheet content increased, and β turn and random coil changed only within a narrow range. These results indicate that the Maillard reaction promoted a transition from a β turn dominated structure to a conformation with higher β sheet content, accompanied by changes in structural flexibility.

### 3.8. SEM Observation of the Effect of Maillard Reaction Time on the Microstructure of Walnut Gluten Peptides

**Figure 9:**
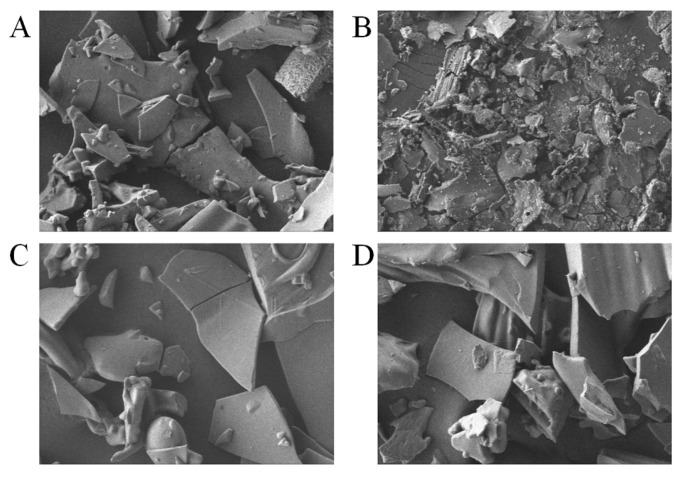
Scanning electron microscope images of walnut gluten peptides at different Maillard reaction times (×500). Scale bar = 20 μm (applies to all panels). (**A**) 0 h; (**B**) 1 h; (**C**) 2 h; (**D**) 3 h.

SEM observations clearly showed that the Maillard reaction markedly altered the aggregated structure of walnut gluten peptides. As shown in Figure 9A, the unmodified sample exhibited a loose, porous, and irregular sheet-like and granular structure with a rough surface and clear boundaries between particles, indicating that the peptide molecules existed in a relatively dispersed state before the Maillard reaction.

With increasing reaction time, the microstructure changed progressively. As shown in Figure 9B, after 1 h of reaction, the initial loose structure began to disappear, surface roughness increased, and preliminary adhesion and fusion appeared, forming a discontinuous porous network. This suggests that early crosslinking and intermolecular interactions, such as hydrophobic interactions, began to drive peptide aggregation.

As shown in Figure 9C, after 2 h the degree of aggregation increased further. Numerous granular aggregates with different sizes were observed, and these aggregates became interconnected, forming a more continuous three-dimensional network with reduced pore size. This morphology corresponds to the intermediate stage of the Maillard reaction, in which extensive peptide sugar crosslinking and hydrophobic aggregation jointly contributed to the formation of a preliminary molecular network [48].

After 3 h, as shown in Figure 9D, the sample exhibited larger and more continuous block-like aggregates with clearer boundaries and relatively smoother surfaces. The original porous network had largely disappeared, and the structure became highly compact. These results indicate that at the late-stage of the Maillard reaction, the crosslinking network became more developed, while stronger intermolecular interactions promoted further folding and close packing of peptide chains, finally leading to the formation of dense aggregates. Overall, the morphological evolution from dispersed and porous structures to dense and continuous aggregates provides direct evidence that covalent and noncovalent interactions were progressively enhanced during the Maillard reaction and gradually dominated structural assembly.

### 3.9. Analysis of Free Amino Acid Composition

As shown in Table 7, the content of free amino acids in walnut gluten peptides changed markedly during the Maillard reaction. Most free amino acids increased sharply after 1 h of reaction, and then decreased rapidly at 2 to 3 h to their initial levels or even lower. For example, the content of L alanine increased from 3178 μg/g at 0 h to 46,989 μg/g at 1 h, representing a 13.8 fold increase. L phenylalanine, L lysine, L threonine, and L tryptophan showed similar trends.

While this increase is likely due to enhanced extractability following glycation-induced conformational loosening, alternative explanations, such as matrix disruption or minor artifacts from the extraction procedure, cannot be completely ruled out. This phenomenon may be attributed to the initial glycation reaction between sugar molecules and nucleophilic groups, such as the ε amino group of lysine residues in the peptide chains, which loosened peptide conformation and exposed amino acid residues that had previously been buried inside the structure. These residues could then be more effectively extracted by sulfosalicylic acid, leading to an apparent increase in the detectable free amino acid content [49]. As the reaction proceeded, free amino groups increasingly participated in the Amadori rearrangement and subsequent polymerization reactions, forming irreversible covalent adducts such as precursors of melanoidin-like products, which led to a marked decrease in detectable free amino acids.

In addition, L arginine, L ornithine, and L glutamine showed a continuous decline throughout the reaction, suggesting that their side-chain functional groups may also have participated in the reaction or become less accessible because of increased steric hindrance. Overall, the dynamic changes in free amino acids reflect the strong effect of the Maillard reaction on peptide surface chemistry and the accessibility of reactive groups.

### 3.10. Analysis of the Dynamic Evolution of Volatile Flavor Compounds Based on GC IMS

GC IMS was used to monitor the dynamic changes in volatile flavor compounds of walnut gluten peptides during the Maillard reaction. A total of 60 volatile compounds were identified, mainly including aldehydes, ketones, esters, furans, pyrazines, and thiazoles.

**Figure 10:**
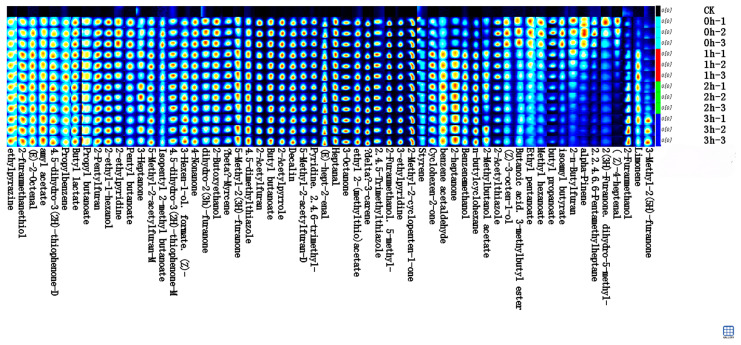
Gas chromatography–ion mobility spectrometry fingerprint profiles of walnut gluten peptides and fructose Maillard products at different reaction times.

As shown in Figure 10, the overall evolution of volatile flavor compounds during the Maillard reaction was clearly reflected by the GC IMS fingerprint spectra. Comparison of the spectra at 0, 1, 2, and 3 h showed the dynamic formation and accumulation of flavor compounds over time. In the fingerprint plots, each row represents a sample and its parallel replicate, and each column corresponds to a specific volatile compound. Color intensity reflects the relative content of each compound, with white to red indicating low to high levels.

At 0 h, the sample showed the simplest volatile profile, with few signal spots and generally weak color intensity, mainly representing the background flavor of the original walnut gluten peptides. After 1 h of reaction, obvious changes appeared, and many new volatile compounds were generated and accumulated. In the fingerprint spectra, darker red signal spots appeared in the middle region and in areas with longer retention times, indicating that the contents of some early and intermediate Maillard reaction products increased markedly. These compounds included aldehydes and furans such as phenylacetaldehyde, which contributes a floral note, and diacetylfuran, which contributes a caramel like aroma.

At 2 h, the formation of volatile flavor compounds reached a high level. The overall fingerprint color became the most intense, and most compound columns appeared deep red, indicating substantial accumulation of volatile components. In particular, pyrazines and sulfur-containing heterocyclic compounds, which contribute strongly to roasted and nutty aromas, were formed in large amounts at this stage, making the flavor profile richer and fuller. At 3 h, the signal intensity of most volatile compounds remained relatively high, indicating that the flavor profile had largely formed and become stable. However, some compound columns became slightly weaker than those at 2 h, suggesting that the reaction had entered a later stage, in which some precursors were depleted and some volatile compounds were further transformed through subsequent reactions.

### 3.11. GC MS Analysis

**Figure 11:**
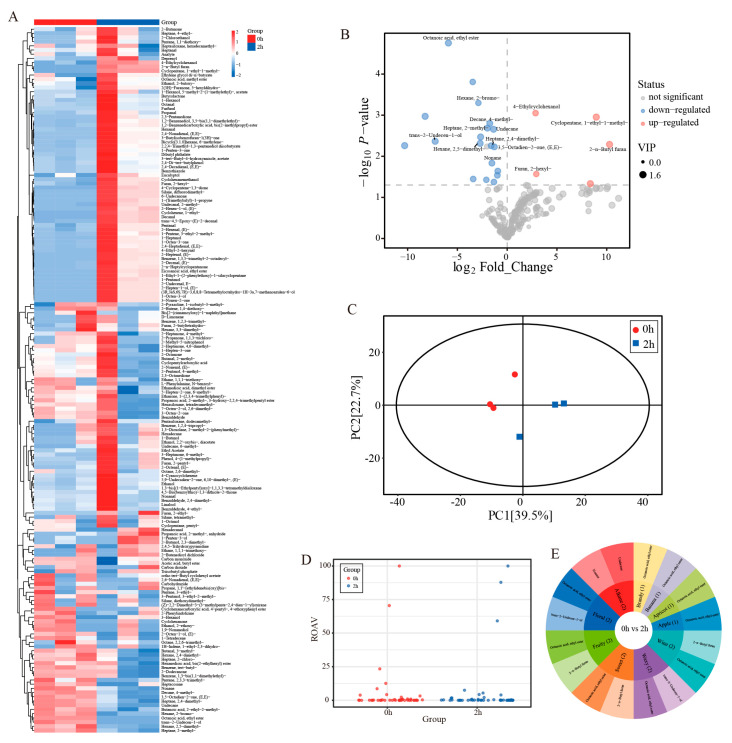
Analysis of differences in flavour compounds before and after the Maillard reaction in walnut gluten peptides based on non-targeted metabolomics, and identification of key aroma compounds ((**A**) Heatmap of cluster analysis at the overall level for all samples. (**B**) Flavour wheel for 0 h versus 2 h (**C**) Volcano plot of metabolites identified as differentially expressed between 0 h and 2 h. (**D**) Scatter plot of ROAV relative odour activity values, (**E**) Scatter plot of PCA model results for 0 h versus 2 h).

Table 8 and Figure 11 show the ROAV distribution of volatile compounds in different groups. Compounds with ROAV ≥ 1 were considered key flavor compounds, whereas those with 0.1 ≤ ROAV < 1 were regarded as modifiers. At 0 h, 10 compounds showed ROAV values above 1. Hexanal made the greatest contribution, with an ROAV of 100, followed by 1 octen 3 one at 70.47 and 1 octen 3 ol at 23.37. At 2 h, the flavor composition changed markedly. 1 Octen 3 one replaced hexanal as the dominant compound, with an ROAV of 100, and the contribution of 1 octen 3 ol increased to 59.05. In addition, trans 2,6 nonadienal changed from a key flavor compound at 0 h, with an ROAV of 2.33, to a modifier at 2 h, whereas 2 pentylfuran and trans 2 heptenal increased from modifiers at 0 h to key flavor compounds at 2 h, with ROAV values of 5.43 and 1.67, respectively. Although benzaldehyde remained a key compound in both groups, its contribution decreased from 12.58 at 0 h to 3.99 at 2 h. These changes indicate that the flavor profile shifted from aldehyde dominance at 0 h to a profile mainly characterized by ketones and alcohols at 2 h.

The principal component analysis results in Figure 11 show that PC1 and PC2 explained 39.5% and 22.7% of the total variance, respectively, with a cumulative contribution of 62.2%. In the PCA score plot, the 0 h samples were tightly clustered with small within-group variation, whereas the 2 h samples were more dispersed. The two groups were clearly separated along PC1, and both remained within the 95% confidence interval, indicating obvious differences in volatile composition between the two stages.

Differential metabolites were further screened by the OPLS DA model using VIP values, *p* values, and fold changes, as shown in Table 8. The volcano plot showed that the downregulated metabolites were more numerous and more concentrated, mainly including alkanes such as 2,5 dimethylhexane, 2 methylheptane, 2,4 dimethylheptane, 2,3,3 trimethylpentane, and nonane. These compounds all had VIP values greater than 1, *p* < 0.05, and log_2_ FC values ranging from negative 1.5 to negative 3.4, indicating marked differences between the two groups. In contrast, the upregulated metabolites were fewer in number but showed larger fold changes, mainly including cyclopentane and furan compounds such as 1 ethyl 1 methylcyclopentane and 2 n butylfuran, which showed strong discriminative ability.

Flavor wheel analysis further showed that ethyl octanoate mainly contributed fruity, sweet, apple, apricot, and brandy-like notes, whereas 2 n butylfuran was associated with wine-like, sweet, and fruity aromas. Differences in the distribution of these compounds between the two groups resulted in a clear separation of the overall flavor profiles.

## 4. Discussion

While the essential amino acid composition was largely retained, this study did not directly measure the nutritional impact of the Maillard reaction. The reaction is known to reduce the bioavailability of certain amino acids, particularly lysine, by converting them into nutritionally unavailable forms [49]. Furthermore, the formation of potentially harmful advanced glycation end-products (AGEs), such as CML and pyrraline, is an inevitable consequence of the Maillard reaction [13]. This is a significant limitation of our study. Future work must quantitatively assess AGEs and conduct in vitro digestibility studies to fully evaluate the nutritional trade-offs of this modification strategy.

## 5. Conclusions

This study successfully established a controlled Maillard reaction modification process for walnut gluten antioxidant peptides. Using response surface methodology, the optimal conditions for maximizing sensory quality and DPPH scavenging activity were a temperature of 78.5 °C, pH of 7.6, and a peptide-to-sugar ratio of 1:1.2 for 2 h, using fructose as the sugar source. The modification induced significant conformational rearrangements in the peptides, including increased β-sheet content and the formation of dense, thermally stable aggregates. These structural changes were directly correlated with the generation of key volatile flavor compounds (e.g., 1-octen-3-one) and enhanced antioxidant activity. However, the study did not assess the formation of potentially harmful advanced glycation end-products or the true bioavailability of amino acids, which are critical areas for future research. Despite these limitations, this work provides a practical strategy and theoretical basis for upgrading walnut processing by-products into functional ingredients with improved flavor and antioxidant properties.

## Figures and Tables

**Table 1:** Factors and Levels in the Response Surface Experimental Design.

Level	A-Temperature/(°C)	B-pH	C-Peptide-to-Sugar Ratio
−1	70	7	1:0.5
0	80	8	1:1
1	90	9	1:1.5

**Table 2:** Evaluation Form.

Evaluation Dimension	Scoring Criteria	Score (Points)
Taste	Rich umami, no salty or bitter taste	9~10
	Pronounced umami, no distinct salty or bitter taste	7~8
	Noticeable umami, slightly salty or bitter taste	5~6
	Weak umami, with salty or bitter taste	3~4
	No distinct umami, pronounced salty or bitter taste	1~2
Aroma	Intense roasted aroma, no off-odor	9~10
	Distinct roasted aroma, no obvious off-odor	7~8
	Identifiable roasted aroma, slightly fishy	5~6
	Faint roasted aroma, distinctly fishy	3~4
	No roasted aroma, Strong fishy odor	1~2

**Table 3:** Testing the coefficients of a regression model for different experimental factors.

Model Items	Sensory Evaluation				DPPH Free Radical Scavenging Rate			
	Mean Square	*F*-Value	*p*-Value	Significance	Mean Square	*F*-Value	*p*-Value	Significance
Model	0.9579	30.34	<0.0001	**	82.83	240.65	<0.0001	**
A-Temperature	0.4	12.67	0.0092	**	22.44	65.2	<0.0001	**
B-pH	0.529	16.76	0.0046	**	9.41	27.34	0.0012	**
C-peptide-to-sugar ratio	1.6	50.68	0.0002	**	197.4	573.53	<0.0001	**
AB	0.045	1.43	0.2714		3.46	10.05	0.0157	*
AC	0.125	3.96	0.0869		0.125	0.3632	0.5658	
BC	0.005	0.1584	0.7025		1.32	3.27	0.1136	
A^2^	0.9645	30.55	0.0009	**	62.31	181.04	<0.0001	**
B^2^	0.3282	10.4	0.0146	*	38.33	111.37	<0.0001	**
C^2^	0.6698	21.22	0.0025	**	65.05	189.01	<0.0001	**
residual	0.0316				0.3442			
missing term	0.0429	12.86	0.0737		0.4695	15.13	0.0632	
pure error	0.0033				0.031			

Note: *p* < 0.05 (*): significant difference; *p* < 0.01 (**): highly significant difference.

**Table 4:** Comprehensive Analysis of R^2^ Values.

Project	*R* ^2^_adj_	*R* ^2^	CV	*R* ^2^_pred_	S/N
Sensory evaluation scores	0.9429	0.9750	2.39	0.7797	16.9141
DPPH radical scavenging rate	0.9926	0.9968	1.01	0.9487	48.0067

**Table 5:** Intermediate products, browning index and colour difference values of fructose-Maillard-modified walnut gluten peptides.

Sample	A294	BI	L*	a*	b*	ΔE*
FMP	1.22 ± 0.01	0.21 ± 0.01	87.00 ± 0.20	3.00 ± 0.20	7.00 ± 0.20	15.07 ± 0.06
WGP	0.77 ± 0.01	0.05 ± 0.01	98.50 ± 0.10	0.20 ± 0.05	0.50 ± 0.05	1.59 ± 0.11

**Table 6:** Comparative analysis of changes in the secondary structure of modified peptides at different reaction times.

Reaction Time	Distribution and Content of Secondary Structures/%			
	α-Helix	β-Sheet	β-Turn	Random Coiling
0 h	21.77 ± 0.45 ^a^	20.08 ± 0.62 ^b^	43.55 ± 0.85 ^a^	14.60 ± 0.38 ^b^
1 h	21.83 ± 0.52 ^a^	19.94 ± 0.58 ^b^	42.62 ± 0.72 ^a,b^	15.61 ± 0.42 ^a,b^
2 h	20.11 ± 0.48 ^b^	21.92 ± 0.64 ^a^	43.01 ± 0.79 ^a^	14.97 ± 0.41 ^b,c^
3 h	20.03 ± 0.43 ^b^	22.57 ± 0.68 ^a^	42.12 ± 0.66 ^b^	15.29 ± 0.37 ^a,c^

Values in the same column with different superscript letters (a–c) are significantly different (*p* < 0.05).

**Table 7:** Free amino acid content of modified peptides at different reaction times.

Amino Acids	0 h	1 h	2 h	3 h
1-Methylhistidine	0.51 ± 0.02 ^b^	2.24 ± 0.07 ^a^	0.41 ± 0.02 ^c^	0.36 ± 0.03 ^c^
3-Methylhistidine	0.70 ± 0.05 ^b^	2.42 ± 0.08 ^a^	0.60 ± 0.03 ^c^	0.57 ± 0.01 ^c^
4-Aminobutanoic Acid (GABA)	6.32 ± 0.39 ^b^	18.75 ± 0.89 ^a^	6.50 ± 0.31 ^b^	6.46 ± 0.34 ^b^
Alanine	3178.08 ± 167.78 ^b^	46,989.23 ± 8769.41 ^a^	1988.89 ± 64.17 ^b^	1771.37 ± 98.68 ^b^
Arginine	1160.76 ± 34.91 ^a^	1061.93 ± 48.35 ^b^	1008.44 ± 42.63 ^b^	934.25 ± 4.99 ^c^
Asparagine	3666.94 ± 275.12 ^b^	5865.65 ± 73.93 ^a^	3675.50 ± 61.17 ^b^	3588.53 ± 215.15 ^b^
Aspartic Acid	141.35 ± 7.81 ^b^	352.65 ± 5.11 ^a^	132.43 ± 5.66 ^b,c^	125.88 ± 9.2 ^c^
Citrulline	48.30 ± 4.07 ^a^	45.57 ± 0.97 ^a^	43.31 ± 0.95 ^a^	43.74 ± 3.5 ^a^
Glutamic Acid	2115.10 ± 124.20 ^b^	3962.37 ± 601.36 ^a^	1934.01 ± 33.95 ^b^	1950.39 ± 109.77 ^b^
Glutamine	1424.89 ± 77.19 ^a^	1270.05 ± 14.72 ^b^	970.49 ± 9.48 ^c^	780.67 ± 29.84 ^d^
Histidine	279.40 ± 10.43 ^b^	760.86 ± 30.62 ^a^	234.23 ± 10.41 ^c^	222 ± 2.42 ^c^
Lysine	236.40 ± 9.89 ^b^	674.42 ± 22.76 ^a^	221.76 ± 13.43 ^b^	209.21 ± 4.06 ^b^
Methionine	257.96 ± 8.43 ^b^	585.97 ± 23.85 ^a^	210.89 ± 7.23 ^c^	200.02 ± 4.59 ^c^
Ornithine	71.74 ± 2.33 ^a^	59.75 ± 1.40 ^b^	41.86 ± 2.57 ^c^	35.15 ± 0.74 ^d^
Phenylalanine	673.71 ± 13.95 ^b^	1932.42 ± 72.32 ^a^	587.89 ± 21.46 ^c^	556.53 ± 7.21 ^c^
Proline	25.86 ± 1.78 ^b^	268.05 ± 51.10 ^a^	18.31 ± 0.31 ^b^	17.73 ± 0.8 ^b^
Threonine	269.54 ± 10.59 ^b^	1058.54 ± 144.88 ^a^	205.65 ± 6.01 ^b^	204.87 ± 15.18 ^b^
Tryptophan	223.01 ± 5.12 ^b^	644.80 ± 28.11 ^a^	210.91 ± 5.77 ^b^	199.36 ± 1.67 ^b^
Tyrosine	245.71 ± 3.71 ^b^	411.14 ± 40.44 ^a^	183.07 ± 10.07 ^c^	176.70 ± 2.23 ^c^
Valine	932.28 ± 25.31 ^b^	2300.82 ± 89.32 ^a^	811.31 ± 31.43 ^c^	793.23 ± 8.53 ^c^
Serine	638.60 ± 23.95 ^b^	1506.98 ± 45.82 ^a^	546.82 ± 18.87 ^c^	516.08 ± 10.92 ^c^

Values in the same column with different superscript letters (a–d) are significantly different (*p* < 0.05).

**Table 8:** ROAV values for key volatile compounds at different reaction times.

Peak	ROAV_Value_of_Group_0 h	ROAV_Value_of_Group_2 h
Propanal	0.14	0.16
Ethyl Acetate	0.03	0.11
Butanal, 2-methyl	0.9	0.32
Ethanol	0.35	0.44
Nonane	0	0
Pentanal	4.24	7.51
1-Penten-3-one	0.04	0.35
2,3-Pentanedione	0	0.06
Acetic acid, butyl ester	0.1	0.02
Hexanal	100	87.82
1-Butanol	0	0.01
Heptanal	3.87	1.81
1-Penten-3-ol	0.01	0.04
Eucalyptol	0.09	0.18
3-Hexanol	0	0
Furan, 2-pentyl	0.91	5.43
Ethanol, 2-ethoxy	0	0
1-Pentanol	0.11	0.46
2-Octanone	0.36	0.16
Octanal	3.24	2.11
Cyclohexanone	0	0
1-Octen-3-one	70.47	100
2-Heptenal, (E)	0.8	1.67
5-Hepten-2-one, 6-methyl	0.06	0.02
1-Hexanol	1.01	0.7
Nonanal	8.61	4.31
2-Octenal, (E)	0.08	0.25
Octanoic acid, ethyl ester	0.02	0
1-Octen-3-ol	23.37	59.05
Furfural	0.01	0
1-Heptanol	0.59	0.85
Decanal	0	0.04
Benzaldehyde	12.58	3.99
6-Undecanone	0	0
Linalool	0.3	0.16
1-Octanol	0.04	0.02
2,6-Nonadienal, (E,E)	2.33	0.25
Benzaldehyde, 4-ethyl	0.01	0.01
Benzothiazole	0.02	0.01
2(3H)-Furanone, 5-hexyldihydro	0.16	0.17
3-Butylisobenzofuran-1(3H)-one	0	0.04

## Data Availability

The original contributions presented in this study are included in the article. Further inquiries can be directed to the corresponding author.
